# What Is the Brain Basis of Intelligence?

**DOI:** 10.1371/journal.pbio.1001078

**Published:** 2011-06-14

**Authors:** Charles F. Stevens

**Affiliations:** The Salk Institute for Biological Studies, La Jolla, California, United States of America

**Figure:**
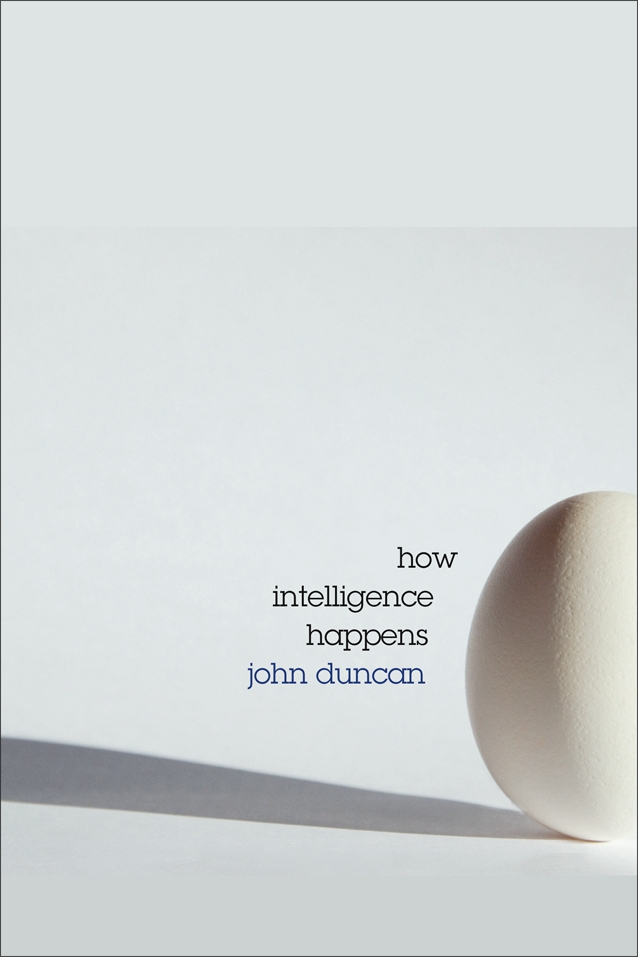
Duncan J (2010) How Intelligence Happens. New Haven, CT: Yale University Press. 256 p. ISBN 978-0300154115 (hardcover). US$28.00.

**About the Author**

Charles F. Stevens, Professor at the Salk Institute, is mainly known for work in the biophysics of ion channels and for studies of the molecular and cell biological mechanisms underlying synaptic transmission. In recent years, he has switched mainly to theoretical neurobiology and is working to identify the design principles that brains use to endow their neural circuits with a scalable architecture—that is, circuits that can be made computationally more powerful by simply making them larger.

Developing civilization arguably would be impossible without human intelligence, as would be our proudest achievements: Shakespeare's plays, Mozart's piano concertos, Darwin's theory of evolution, and Einstein's relativity. What is it about the human brain that endows us with the intelligence that provides the basis of such achievements? John Duncan sets out to answer this question in his new book *How Intelligence Happens*.

Duncan, Assistant Director of the MRC Cognition and Brain Sciences Unit in Cambridge (United Kingdom), has brought together material from psychology, neuropsychology, cognitive science, artificial intelligence, brain imaging, and single-cell recording studies to develop a theory of human intelligence. The book is intended for a lay audience—and thus is written in nontechnical language with relaxed, anecdotal sections and personal stories interspersed with descriptions of experimental results—but it will be enjoyed by professionals who want to learn about frontal lobe function and modern ideas of human intelligence.

Briefly, Duncan's main argument is that our frontal lobes contain circuits that break complex problems down into manageable subproblems, and then oversee the solution of these subproblems in the proper order. This argument is developed in five chapters, the first of which introduces the idea of general (or fluid) intelligence in a historical context by focusing on Spearman's research, which gave rise to his notion of *g* (general) and *s* (special) factors. Each kind of task, such as remembering the words in a list or figuring out a puzzle, depends on special factors (*s*) needed to make you good at that task and a general factor (*g*) that helps you to be good at any task.

After presenting basic neuroanatomy and describing some methods for studying brain function (for example, functional magnetic resonance imaging, fMRI), Duncan recounts how he had the insight that fluid intelligence resides mostly in the frontal lobes. When he was a postdoctoral fellow, one of Duncan's jobs was to do psychological testing on candidate bus drivers to try to predict which ones were more likely to have accidents. During this testing, he noticed that drivers with low *g* scores knew the rules they were supposed to follow in performing the tests, but somehow could not manage to apply these rules effectively. Their behavior reminded Duncan of Luria's descriptions of disorganized behavior exhibited by patients with frontal lobe lesions, an observation that set Duncan's future career path.

Drawing on observations from artificial intelligence, a field whose goal is to give computers human-like intelligence, Duncan argues that, although brains and digital computers have quite different architectures and use different methods to carry out their computations, problem solving by computers offers a good model for how people do it: you have to break down the entire problem into small subproblems, and solve them in sequence. Being able to execute this strategy is at the heart of fluid intelligence, and its neuronal substrate should therefore be found in the frontal lobes. But how do the neurons in the frontal lobes do it? Duncan reviews recent research showing that circuits in the frontal lobes have the flexibility to encode, in the firing rate of resident neurons, just about anything being held in mind. The firing of a particular neuron might record a location that is to be held in short-term memory at one time, or be a cat-versus-dog classifier at another time.

One of the things that makes it hard for artificial intelligence to mimic human intelligence is that we can be, but often are not, rational beings. Capturing our special forms of irrationality poses a special problem for computer programmers. In the final chapter, Duncan describes some ongoing and incomplete research to give an impression of the direction intelligence studies is heading. For example, he outlines some experiments that permit us to “read a person's mind” by examining the pattern of brain activity revealed by fMRI.

The experimental findings relating to human intelligence often are unexpected and arresting—for example, people prefer a medical treatment when they are told it saves the lives of 90% of the patients over the same treatment when told that 10% of the patients die. The opportunity to learn about these discoveries will make this book rewarding for the lay reader. At the same time, the broad range of disciplines represented will provide many professional neurobiologists with welcome new facts and ideas.

